# La perspectiva salud-ambiente como herramienta para la ecofarmacovigilancia

**DOI:** 10.15446/rsap.V27n1.111296

**Published:** 2025-01-01

**Authors:** Diego Mauricio Quijano-Prieto, José Gilberto Orozco-Díaz

**Affiliations:** 1 DQ: Ing. Ambiental y Sanitario. M. Sc. Medio Ambiente y Desarrollo. Ph. D.(c) Salud Pública. Investigador. Bogotá, Colombia. dmquijanop@unal.edu.co Universidad Nacional de Colombia Bogotá Colombia dmquijanop@unal.edu.co; 2 JO: MD. Esp. Epidemiología. M. Sc. Ciencias Farmacología. Ph. D. Salud Pública. Universidad Nacional de Colombia. Bogotá, Colombia. jgorozcod@unal.edu.co Universidad Nacional de Colombia Universidad Nacional de Colombia Bogotá Colombia jgorozcod@unal.edu.co

**Keywords:** Ambiente, preparaciones farmacéuticas, contaminación ambiental, salud pública, medio ambiente y salud pública, salud ambiental *(fuente: DeCS, BIREME)*, Environment, pharmaceutical preparations, environmental pollution, public health, environment and public health, environmental health *(source: MeSH, NLM)*

## Abstract

La contaminación ambiental por medicamentos es un problema ambiental emergente y una preocupación de salud pública. Se han detectado fármacos en diversos ecosistemas, donde han causado impactos negativos, y dadas sus propiedades bioactivas se consideran potencialmente dañinos para diversas especies. La ecofarmacovigilancia estudia la detección, la evaluación, la comprensión y la prevención de los efectos adversos relacionados con la presencia de fármacos en el ambiente. El presente ensayo analiza la ecofarmacovigilancia mediante un paralelo con el libro Primavera silenciosa, se resaltan vacíos de conocimiento y se aborda la problemática con un enfoque que destaca aspectos sociales de los problemas de salud-enfermedad-cuidado. Se señala que el abordaje actual de la ecofarmacovigilancia pierde de vista el papel determinante de las relaciones sociales y se enfoca en la búsqueda de causas inmediatas, en el corto plazo y en el estudio de factores aislados. Se observa la necesidad de un replanteamiento teórico del problema, donde se resalten las múltiples relaciones ecosociales. En la actualidad, como sociedad, se está ante la posibilidad de conocer, replantear y tomar acciones frente a la forma en que los seres humanos se relacionan entre sí y con la naturaleza y cómo se asumen las cuestiones de salud-enfermedad-cuidado.

## Abstract (en)

Environmental pollution by pharmaceutical is an emerging environmental issue and a public health concern. Active pharmaceutical ingredients have been detected in various ecosystems, where they have caused negative impacts, and considering their bioactive properties, they are considered potentially harmful to several species. Ecopharmacovigilance is concerned with the detection, evaluation, understanding, and prevention of adverse effects related to the presence of pharmaceuticals in the environment. This essay analyzes ecopharmacovigilance through a parallel with the book Silent spring, highlighting gaps in knowledge and addressing them with an approach that focuses on social aspects of health-disease-care problems. The present document points out that the current approach to ecopharmacovigilance loses sight of the determining role of social relationships, and focuses on the short-term, the study of isolated factors, and the search for immediate causes. There is a need for a theoretical rethinking of the problem is referred to, where the multiple ecosocial relationships are highlighted. Currently, the society has the possibility of knowing, rethinking, and taking action regarding how humans deal with each other and nature, in relation to health-disease-care issues.

La presencia de principios activos de medicamentos (PA) en los ecosistemas, sus efectos negativos sobre diversas especies y el riesgo para la salud humana son una problemática de interés a nivel mundial; la ecofarmacovigilancia estudia dicha problemática 1.

Los PA son considerados contaminantes emergentes debido al peligro potencial que representan para la salud humana y el ambiente, a que no están incluidos en los programas de vigilancia y no existe una normativa específica para ellos 2; se consideran contaminantes ubicuos, dada su presencia en diversas matrices ambientales y en animales no medicados 3. Se prevé un aumento del consumo mundial de fármacos, así como un incremento en su presencia en los ecosistemas, como consecuencia del aumento de la población mundial; el tratamiento de enfermedades crónicas y aquellas relacionadas con el envejecimiento; cambios en la práctica clínica; una mayor medicalización y un consumo exorbitante promovido por los ingresos por venta de medicamentos en la industria farmacéutica; el crecimiento económico; el aumento global del consumo de productos animales y el cambio hacia prácticas agrícolas más intensivas 4,5.

La Comisión Europea declaró que la contaminación de las aguas y los suelos con residuos farmacéuticos es un problema ambiental emergente y de salud pública 1.

## Un problema que no se anticipó

"Se produjo una extraña quietud. Los pájaros, por ejemplo... ¿dónde habían ido?... Las pocas aves que se veían se hallaban moribundas: temblaban violentamente y no podían volar. Era una primavera sin voces" (6, p. 18).

En 1962 se publicó Primavera silenciosa, obra de Rachel Carson, que expuso los impactos ambientales negativos debido a los pesticidas organoclorados. La publicación suscitó críticas que trataron de desacreditar lo denunciado, sin embargo, el mensaje de Carson causó agitación y promovió, en conjunto con el cúmulo de evidencia científica y el movimiento social, la prohibición del dicloro dife-nil tricloroetano (DDT) en Estados Unidos, lo cual sentó un precedente sobre los temas ambientales 7.

De manera similar a como comenzaron a desaparecer las aves del relato de Carson, en la década de 1990 la población de buitres del subcontinente indio fue diezmada por razones desconocidas en ese momento. Fue hasta el año 2004, posteriormente al deceso de decenas de millones de buitres 8 y después de diversas indagaciones to-xicológicas, que se identificó que el diclofenaco contenido en los cadáveres de ganado, previamente tratados con este medicamento, era el responsable del declive de la población de tres especies de buitres *(Gyps bengalensis, Gyps in-dicus y Gyps tenuirostris)*^9^. Esto condujo al retiro de la licencia de fabricación de diclofenaco para uso veterinario en 2006 en India, seguido por Nepal y Pakistán 10.

Esta catástrofe ecológica no fue prevista por pruebas de laboratorio, en parte porque no se cuenta con el conocimiento, el tiempo y el dinero para realizar pruebas sobre todas las especies y todos los PA; en consecuencia, no se pueden descartar otras sorpresas en el futuro, por lo que parece prudente reducir la entrada y presencia de PA al ambiente 3.

Para algunos autores, "la extinción de los buitres puede tener implicaciones sociales, económicas, ecológicas y de salud pública de gran alcance" (11, p. 4), dado el papel que cumplen estas aves rapaces en el control de enfermedades del ganado, así como la posible ocupación del nicho de los buitres por otras especies, con un potencial efecto nocivo para la salud humana, debido a su función para la eliminación de cadáveres. A ello se suma la alteración de los aspectos sociales, por la importancia de los buitres en la cultura hindú 10,11. Lo anterior muestra que los efectos sobre especies no se limitan a aspectos puntuales, estos tienen múltiples ramificaciones por las relaciones de interdependencia.

Numerosos estudios informan sobre efectos adversos de los PA en las concentraciones encontradas en los ecosistemas, entre ellos, afectación de fauna estercolera por ivermectina 12; efectos negativos en el comportamiento y la tasa de alimentación en *Perca fluviatilis* por benzodiazepinas 13; fitotoxicidad; afectaciones en abundancia y diversidad de la comunidad microbiana del suelo debido a enrofloxacina y otros antibióticos de uso veterinario; 14 y alteraciones sexuales por estrógenos sintéticos o naturales en poblaciones de peces, lo que afecta tanto la dinámica como el éxito reproductivo y suscita importantes preocupaciones sobre impactos en la vida silvestre por la exposición a largo plazo a bajas concentraciones de disruptores endocrinos estrogénicos 12.

Preocupan también los efectos asociados al consumo de antibióticos, ya que su exagerado uso se relaciona con contaminación ambiental, además de la selección y la dispersión de bacterias resistentes a los antibióticos, cuyos impactos se reconocen como una preocupación a escala mundial 15.

A pesar de la creciente evidencia sobre los riesgos para la salud ambiental y humana, existen vacíos importantes en torno al conocimiento de la problemática. Los riesgos ecológicos podrían ser mayores de lo previsto por las pruebas realizadas para PA individualmente, a causa de las interacciones toxicológicas de las posibles mezclas, los efectos en el largo plazo y los efectos indirectos, los cuales no son fáciles de prever, dado que los efectos observados en el ambiente son de difícil atribución a un compuesto específico o a sus probables combinaciones, considerando la posibilidad de traslape de propiedades de dichas sustancias y la complejidad de los ecosistemas 12.

## Un problema que no parece ser tan importante

"Estamos acostumbrados a buscar los grandes e inmediatos efectos e ignorar todo lo demás. A menos que estos aparezcan rápidamente y en forma tan obvia que no puedan ser ignorados, negamos la existencia del peligro".

De acuerdo con la Comisión Europea, "hasta ahora, no se ha establecido un vínculo claro entre los productos farmacéuticos presentes en el medio ambiente y los efectos directos en la salud humana" (16, p. 4). Para la Organización Mundial de la Salud (OMS), debido a que las concentraciones determinadas en agua potable normalmente están por debajo de las dosis terapéuticas mínimas, es poco probable que supongan una amenaza para la salud humana. Sin embargo, las dos instituciones consideran importante adoptar un enfoque de precaución 16. Otros autores señalan que "no hay justificación para considerar los productos farmacéuticos como una clase de compuestos como un problema" (17, p. 99), ya que estos son simplemente un subconjunto de contaminantes emergentes y es necesario abordarlos caso por caso 17.

Para algunos investigadores, se requiere más información para tener una imagen clara del problema, que permita tomar decisiones acertadas con respecto a la salud humana y ambiental, ya que sería imprudente afirmar que los PA están causando un efecto ambiental significativo, sin evidencia concluyente. Estos autores destacan también que "Al evaluar productos farmacéuticos, los beneficios para la salud humana deben tener prioridad sobre cualquier daño ambiental potencial" (18, p. 769), lo cual demuestra una marcada tendencia antropocéntrica al evaluar los problemas ambientales y plantea una negación de la interdependencia humano-ambiente. Adicionalmente, se hace pertinente preguntarse si se requiere demostrar que los medicamentos causan daño o demostrar que no lo hacen, ya que ninguna de las dos situaciones está plenamente demostrada, y quizá nunca se llegue a ello, por tanto, lo procedente sería aplicar el principio de precaución.

Aunque para algunos actores la problemática de los PA no es un tema urgente, se han propuesto estrategias orientadas a intervenir el ciclo de vida del medicamento para minimizar la presencia de productos farmacéuticos en el ambiente, entre las que se encuentran: tratamientos avanzados de aguas residuales; medidas al "final del tubo", como tratamiento de aguas residuales de producción de medicamentos, hospitales, rellenos sanitarios y sistemas cerrados en acuicultura; eliminación adecuada de medicamentos; disminución del uso; administración de menores dosis y aumento del bienestar humano y animal con intervenciones transectoriales 19.

Una de las alternativas legislativas vigentes en Estados Unidos, Canadá y los países miembros de la Unión Europea es la Evaluación del Riesgo Ambiental (ERA), una herramienta cuyo propósito es evaluar, prevenir y limitar potenciales efectos ambientales adversos debido a los productos farmacéuticos, tanto de uso humano como veterinario. La ERA es un requisito legal para la autorización de mercadeo de un nuevo medicamento, sin embargo, el impacto potencial no constituye un criterio para limitar su comercialización 20.

Como se observa en lo presentado y en concordancia con lo que describe Carson, no se emprenden acciones concretas hasta que no se dispone de una evidencia contundente o efectos negativos abrumadores; de lo contrario, no se presta suficiente atención al peligro. Otros autores consideran que el problema tiene una mayor relevancia.

## Un problema que sí parece ser importante

"Sólo en el momento representado por el siglo actual una especie, el hombre, adquirió un poder significativo para alterar la naturaleza de su mundo" (6, p. 19).

La Unión Europea sostiene que los residuos farmacéuticos se han convertido en una fuente importante de contaminación ambiental, en parte porque estos productos están diseñados para no degradarse, o hacerlo lentamente, de manera que se facilite su comercialización, por lo que representan un riesgo, ya que ingresan, persisten y se diseminan en los ecosistemas. Asimismo, señala que "hay suficientes pruebas que justifican la necesidad de adoptar medidas para reducir los riesgos ligados a la presencia de productos farmacéuticos en el medio ambiente" (16, p. 14).

Las concentraciones de PA en el agua generalmente están en el rango de ng/L a /ng/L 21, lo cual implica que no deberían esperarse efectos farmacológicos inmediatos sobre la población expuesta; sin embargo, no se ha estudiado el posible efecto sobre la salud pública de la exposición a largo plazo, por lo que no puede considerarse inofensiva, especialmente teniendo en cuenta que los productos farmacéuticos son fabricados con la intención de que sean bioactivos, por lo que son potencialmente dañinos para la flora y la fauna acuáticas. De acuerdo con el principio de precaución, las autoridades de salud pública deben tomar medidas para evitar la contaminación del agua 22.

Se plantea que deben investigarse los efectos secundarios sobre la salud humana, acuática y animal, mediante estudios toxicológicos y de seguridad exhaustivos, y que se requieren esfuerzos para reducir el problema, junto con algunas regulaciones adecuadas para monitorearlos y controlarlos 23; temas definitivamente importantes que, sin embargo, no abarcan los aspectos relacionales y complejos de la manera de entender los problemas de salud-enfermedad-cuidado, ni la profunda relación sociedad-naturaleza, como se ahondará más adelante.

La OMS ha informado que, "en todo el mundo, más del 50% de todos los medicamentos se recetan, dispensan o venden de manera inapropiada, mientras que el 50% de los pacientes no los toman correctamente"; adicionalmente, reconoce los "motivos de lucro por la venta de medicamentos" como una de las razones por las cuales los medicamentos se usan irracionalmente (24, p. 1). De lo anterior se colige la posibilidad de abordajes educativos, normativos y gerenciales frente a los problemas de prescripción, dispensación y administración 24; además, se entrevé un trasfondo de intereses económicos como parte del origen del problema.

Entre los posibles problemas de salud derivados de la presencia de PA en el agua potable se encuentran un mayor riesgo de desarrollar cáncer, discapacidad reproductiva y selección y desarrollo de bacterias resistentes a los antibióticos 25. Lo anterior, centrando la atención en los medicamentos quimioterapéuticos u hormonas que pueden ser cancerígenas, aquellas que pueden obstaculizar la reproducción o el desarrollo y antibióticos que pueden permitir que las bacterias muten en formas más peligrosas 26, asunto que la OMS ha declarado como una de las diez principales amenazas de salud pública a las que se enfrenta la humanidad 15.

Es dudoso que incluso la mejor tecnología pueda predecir el impacto a largo plazo de la exposición continua a combinaciones de PA en organismos altamente complejos. Si la exposición a concentraciones bajas de sustancias como los estrógenos exógenos representa una amenaza para la salud humana, la reducción de la exposición brindará una oportunidad para la prevención primaria 25.

Tanto quienes consideran que el problema parece ser importante como quienes no están de acuerdo con tal postura, proponen formas de entenderlo y abordarlo desde la lógica causa-efecto, a partir de propuestas para evitar que dichas moléculas alcancen los ecosistemas o mediante disposiciones tecnológicas o educativas enfocadas en el denominado ciclo de vida del medicamento. Sin embargo, no se trata solo de la presencia de moléculas depositadas en los ecosistemas de manera espontánea, natural o inevitable, sino de una compleja red de interrelaciones entre acciones humanas y ecosistemas, que dan cuenta de esa gran capacidad que ha adquirido el ser humano para alterar de manera significativa la naturaleza.

## La perspectiva salud-ambiente como herramienta para abordar la ecofarmacovigilancia

"Nos encontramos ahora en una encrucijada. Pero al revés de los caminos del poema de Robert Frost, ambos no son igualmente bellos. El que hemos estado siguiendo es de una facilidad que decepciona, una carretera de primerísimo orden por la que progresamos a gran velocidad, pero a cuyo fin está el desastre. El otro recodo -el camino "menos frecuentado"- ofrece al final nuestra única oportunidad para alcanzar una meta que asegure la conservación de nuestra tierra" (6, p. 213).

El paralelo presentado entre Primavera silenciosa y la problemática de los PA en los ecosistemas se utiliza como herramienta discursiva, ya que, claramente, los medicamentos y los plaguicidas distan de ser iguales, de la misma manera que los efectos de unos y otros sobre el ambiente. Sin embargo, valiéndose de esta referencia, el presente ensayo invita a la reflexión sobre una problemática que merece ser profundizada y analizada con detenimiento.

Así como la preocupación por los efectos no deseados de los plaguicidas, el interés, los estudios, los hallazgos y las medidas para abordar la presencia de los PA en el ambiente y sus efectos negativos se dan en un contexto de preocupación generalizada por los efectos de las actividades humanas sobre la naturaleza, incluida la propia humanidad. Sumado a esto, el refinamiento de las técnicas de medición ha permitido la detección de una amplia variedad de PA en las matrices ambientales. La literatura académica y gris reconoce un vacío de conocimiento sobre la problemática y se advierte una tendencia a abordarla desde medidas tecnológicas, educativas, gerenciales y normativas, con una visión que desvía la atención de los problemas fundamentales de la salud pública, ya que no es fácil encontrar una reflexión que señale la relación de los procesos sociales con este asunto.

Se observa que, tanto quienes consideran que no es un problema importante como quienes sí lo reconocen como tal, posicionan un abordaje desde la toxicidad de las moléculas y a través de estrategias orientadas a intervenir el ciclo de vida del medicamento. Adicionalmente, se percibe un marcado enfoque antropocéntrico, ya que se enfatiza en los efectos negativos que los PA puedan generar en humanos. El enfoque de estudio actual de la ecofarmacovigilancia pierde de vista el papel determinante de las relaciones sociales y se enfoca en la búsqueda de causas inmediatas, en el corto plazo y en el estudio de factores aislados. De manera particular, se enfoca en lo que se ha definido en las publicaciones científicas como causas (descargas de aguas residuales, falta de medidas legislativas, entre otras), las cuales no consideran las relaciones con los procesos estructurales que las originan; por ende, se requiere reenfocar la investigación y las prácticas, para superar la lógica empírico-analítica y el causalismo con que se aborda actualmente la cuestión.

Desde otro enfoque, es pertinente propender por un modelo de investigación y desarrollo de medicamentos basado en intereses sanitarios más que comerciales. Es también oportuno cuestionar si la principal alternativa para los problemas de salud debe ser el uso de medicamentos, especialmente si el principal dinamizador de la investigación, el desarrollo, la producción y el uso de medicamentos no son los problemas o los beneficios en salud, sino la rentabilidad 27.

Los medicamentos tienen gran importancia material y simbólica 28. La forma en la que se producen, distribuyen y consumen es un reflejo de las políticas de salud, la política social general y el modelo de desarrollo. Una sociedad medicalizada desatiende las potencialidades del autocuidado y se enfoca en el consumismo, favoreciendo con ello la lógica de mercado sobre los modos de vivir; y es un problema que permite ver la subsunción del consumo en salud al capital.

Si bien parece sensato continuar investigando como se viene haciendo, entre otras razones porque no se cuenta con datos de seguimiento a largo plazo ni de las interacciones de la diversidad de sustancias, parece también necesario hacer un cambio en la aproximación epistemológica que se realiza, ya que no hay que perder de vista el propósito de transformar a través del conocimiento.

Hay una interdependencia entre cómo se observa la realidad, cómo se piensa y cómo se actúa en esta, por lo cual, si se observa a la naturaleza como algo externo, ajeno, como una fuente de suministros o un sumidero de residuos, se estará recortando y dejando de ver una parte del problema. Si, adicionalmente, se ocultan o ignoran las relaciones sociales precursoras de la problemática, se pierde de vista su origen. Es fundamental señalar que se requiere un replanteamiento teórico del problema, entendiendo que tanto métodos como técnicas se subordinan a una lógica analítica de los procesos como conjunto 30.

Si se analiza la problemática fuera de su contexto social, el cual es determinante en su desarrollo, las prácticas derivadas para la intervención tendrán mayores sesgos y limitaciones, teniendo en cuenta que los problemas ambientales radican en la manera como los seres humanos se relacionan con los ecosistemas.

Esta problemática no debe reducirse a analizar los efectos sobre especies, ya que como lo mencionó Rachel Carson, "en la naturaleza nada existe solo" (6, p. 51), y como se mostró con el caso de los buitres en el subcontinente indio, en el cual se observan las múltiples relaciones ecológicas y sociales, el abordaje de la problemática tampoco debe limitarse a la detección de sustancias en los ecosistemas. Se deben explorar las relaciones sociales que dan origen a la problemática.

Como reflexión final y así como los mencionados caminos de Frost y Carson, hoy la sociedad está ante la posibilidad de conocer, replantear y tomar acciones frente a la forma en que los seres humanos se relacionan con la naturaleza y frente a cómo se asumen las cuestiones de salud-enfermedad-cuidado. Se debe propender por relaciones armónicas entre los seres humanos como parte de la naturaleza y se debe trabajar por una salud construida desde el bienestar en estas relaciones ♦
